# Advancing screening for cognitive impairment: the memtrax continuous recognition test

**DOI:** 10.18632/aging.204828

**Published:** 2023-06-14

**Authors:** J. Wesson Ashford, James O. Clifford, Michael F. Bergeron

**Affiliations:** 1 Department of Psychiatry and Behavioral Sciences and Alzheimer’s Center, Stanford University, Stanford, CA 94305, USA; 2 War Related Illness and Injury Study Center and Alzheimer’s Center, VA Palo Alto Health Care System, Palo Alto, CA 94304, USA; 3 Department of Psychology, College of San Mateo, San Mateo, CA 94402, USA; 4 Department of Health Sciences, University of Hartford, West Hartford, CT 06117, USA

**Keywords:** Alzheimer’s disease, memory, online testing, response time, continuous recognition test

Extensive efforts to find a treatment for Alzheimer’s disease (AD) span over 40 years, with the often-repeated request for better means to assess the principal dysfunction of this disease, memory impairment [1, 2]. Tremendous costs and resources have already been consumed in the development of treatments for this prevalent and well recognized condition, e.g., over $40 billion [3]. And the pervasive failures, support the urgent need for instruments far superior to those used even in recent studies [4]. The critical impairment in AD is a disorder of neuroplasticity [5].Thus, cognitive tests which can rapidly, sensitively, frequently, inexpensively, and precisely measure the aspects of memory specifically attacked by AD are principally needed [6].

The memory failure in Alzheimer’s disease initially presents as an impairment of the encoding of new information. This failure is a consequence of a specific disorder of neuroplasticity evidenced by the imbalance of synaptogenesis (making new synapses) and synaptolysis (removing synapses that no longer represent the individual’s memory vector). Substantial evidence suggests that this diathesis is related to dysregulation of the alpha and beta cleavages of the amyloid preprotein (APP) during the processing of novel sensory stimuli and aberrant gamma cleavage of the beta product, though influence of the apo-lipo-protein-E (APOE) is also centrally related. The abnormal processing of the APP stimulates formation of hyperphosphorylated microtubule-associated protein-tau, leading to production of neuropil threads which amputate the neuronal process, both axons and dendrites, in turn causing loss of stored information (see [5] for discussion). Accordingly, a continuous recognition test of memory (CRT; MemTrax) was developed to quickly and accurately quantify memory processing, storage, and rate of retrieval [7]. MemTrax (http://www.memtrax.com) provides 25 unique images with extensive complex information that can be easily encoded by cognizant adults; the healthy human nervous system can readily recognize the 25 repeated images. With a of total 50 images and each shown up to 3 seconds or until a response is made, the whole test takes about 90 seconds. Notably, the AD impairment of complex perception and encoding leads to an inaccuracy and slowing of recognition of the recently presented information. Therefore, MemTrax quickly demonstrates and quantifies the pertinent memory dysfunction of AD.

In the study of MemTrax dynamics, initially, the concepts of “signal detection theory” (SDT) were considered, with the view that each new image represented a signal, and the recognition represented a detection. SDT suggests that there would be a d’ (d-prime) factor which represents the ability to distinguish a new image and repeated image, whereas a beta factor would represent the tendency to favor responding or not responding to a repeated image. However, the data from MemTrax, instead, show that recognition responses (HITs) and Correct Rejections accuracy were not related. Thus, these two variables represent different processes, with HITs representing successful recognitions, a temporal lobe function, while Correct Rejections represents inhibitory processing, which is more related to frontal lobe function.

The second notable finding of this study was that the distribution of recognition response times (RTs) could be explained precisely by a “reverse exponential” function (RevEx, a 2-parameter function), better than the traditional exponential-Gaussian function (ex-Gaussian, a 3-parameter function). With this finding, a new conception of recognition is apparent that is related to the massive processing conducted by large cortical regions. For each specific time-step (about 100 milliseconds) of increased speed without loss of accuracy, a doubling of neural resources is required. Conversely, as slowing occurs, each specific time-step of slowing is associated with a 50% loss of neural resources. This conceptualization can be tested with respect to the loss of synapses in the Alzheimer brain, the factor most associated with loss of cognition in AD (see [5] for discussion). Given that HITs are related to RTs, the RT apparently reflects the processing speed of information being analyzed in the temporal lobes. We hypothesize that further study will reveal that the variability of RTs for an individual will reflect attention and correspond to the proportion of correct-rejections, and thus frontal lobe function. This additional analysis is being investigated with the MemTrax data.

A single administration of MemTrax provides at least as much information as the Montreal Cognitive Assessment (MoCA) [7]. Further, with Machine Learning, distinction of mild cognitive impairment can be replaced by precise assessment of a subject’s performance both along the continua of impairments and the effects of diverse neurodegenerative processes [7, 8]. For monitoring change over time, with over 3,000 images, MemTrax can be repeated frequently (120 tests can be given before a single image is reshown). Moreover, the random selection of categories and images in each of 5 categories provides a completely novel experience for all tests, even if taken multiple times in a single day. Additionally, with “Google Translate”, MemTrax is available in 120 different languages, since only the instructions need translation. Of international importance, cultural variations can present barriers, and as an example of managing this concern, MemTrax is available in China, which provides 5,000 unique images chosen for that culture (http://www.memtrax.com.cn). Note that MemTrax is willingly repeated multiple times (as numerous subjects have taken MemTrax over 1,000 times without request or coercion). Mathematically, the standard error of the estimated memory functions for a subject drop with the square-root of the number of times the test is taken. So, highly reliable estimates of a subject’s memory functions are provided by MemTrax.

The precision of MemTrax would best improve the specification of the severity of cognitive impairment in early phases of Alzheimer’s disease, a period of this disease when paper and pencil and historical recollection only provide poor estimates of function [4]. Further, MemTrax can precisely assess the rate of change over time with repeat testing. By assessing performance metrics and rate of recognition response, MemTrax can screen for many varieties of cognitive impairment and thus would be an ideal tool for use in the elderly US population for the Medicare Annual Wellness Visit [2]. With a test such as MemTrax or other effective online testing, populations can be broadly and inexpensively assessed for AD-related cognitive impairment and then brought into clinical studies to determine what environmental, genetic, or interventional remedies can prevent further development of AD and the pace and/or extent of cognitive decline. MemTrax is especially well suited for assessment of very early AD, including early mild cognitive impairment, a time when the focus should be on prevention of AD pathology, not removal of AD pathology.
